# Procoagulant and Anticoagulant Factors in Childhood Hypothyroidism

**DOI:** 10.1155/2012/156854

**Published:** 2012-07-03

**Authors:** Nevin Kilic, Yildiz Dallar, Enver Simsek, Sirma Karamercan, Ayse Esra Tapci, Bulent Alioglu

**Affiliations:** ^1^Department of Pediatrics, Ankara Training and Research Hospital, The Ministry of Health of Turkey, Ulucanlar Caddesi, Altindag, 06340 Ankara, Turkey; ^2^Department of Pediatric Endocrinology, Ankara Training and Research Hospital, The Ministry of Health of Turkey, Ulucanlar Caddesi, Altindag, 06340 Ankara, Turkey; ^3^Department of Pediatric Hematology, Ankara Training and Research Hospital, The Ministry of Health of Turkey, Ulucanlar Caddesi, Altindag, 06340 Ankara, Turkey

## Abstract

The aim of this study was to investigate the effects of thyroid hormone deficiencies in childhood on the elements of coagulation proteins. Consecutive 54 children with hypothyroidism and 55 healthy controls aged 1 month–16 years were enrolled. One year after Na-L-thyroxine treatment, the study parameters were reevaluated. Thyroid function tests, procoagulant and anticoagulant proteins were performed for children with hypothyroidism and healthy controls. Significant decreased results were found in children with hypothyroidism in terms of fibrinogen, TT, and anticoagulant proteins including AT, PC, PS, and fPS. Significant increases were found with respect to APTT, fibrinogen, and TT. In the evaluation of posttreatment changes a statistically significant increase was found in vWF, FVIII, AT, PC, PS, and fPS. A positive correlation was found between fT4 and vWF, FVIII, PC, and PS. We would like to emphasize that the coagulation system especially vWF and FVIII, and particularly the anticoagulant system, should be monitored closely in patients followed up for hypothyroidism. Thyroid hormones should be examined and, if necessary, hormone replacement therapy should be administered in patients followed up for a predisposition to coagulation. Additionally, further studies with larger series are needed to investigate the effects of hypothyroidism on the coagulation system.

## 1. Introduction

 Previous studies have demonstrated the association between hypothyroidism and cardiovascular disease due to increased atherosclerosis and associated morbidity [1, 2]. Even though the predisposition of hypothyroid patients to bleeding is described, recent studies have shown that insufficient concentration of thyroid hormones induced a hypercoagulable state.

The aim of this study was to investigate the effects of thyroid hormone deficiencies in childhood on the elements of coagulation including procoagulant and anticoagulant proteins. 

## 2. Materials and Methods

### 2.1. Selection of the Study Groups

 Consecutive 54 children with hypothyroidism aged 1 month–16 years who presented for the first time to our hospital between 2008 and 2009 and who were diagnosed with the disease were enrolled in the study. The control group consisted of 55 children of the same age who had no chronic diseases and who had presented to the pediatric outpatient clinic for growth, immunization, and development followup or counseling.

 Data recorded included the chronological age, anthropometric features, presence of concomitant diseases, and medication history of both the patient and control groups. None of the patients had any concomitant disease except for hypothyroidism. Neither the patients nor the controls were on drugs affecting the lipid metabolism and/or homeostasis.

The diagnosis of primary hypothyroidism was established by a high serum TSH (>5 mIU/mL) concentration and a low serum-free T4 concentration according to age reference limits. Patients with a high serum TSH concentration and a normal serum free T4 concentration were diagnosed as having subclinical hypothyroidism. Central hypothyroidism was diagnosed by a low free serum T4 concentration and a serum TSH concentration that is not appropriately elevated. All of the patients had hypothyroidism. One year after Na-L-thyroxine treatment, the study parameters were reevaluated patients diagnosed with hypothyroidism.

### 2.2. Materials and Laboratory Methods

 Thyroid function tests were performed (TSH, TT4, fT4). Prothrombin time (PT) international normalized ratio (INR), activated partial thromboplastin time (APTT), fibrinogen, thrombin time (TT), factor (F) II, FV, FVII, FVIII, FIX, FX, FXI, and von Willebrand's factor (vWF), antithrombin (AT), protein C (PC), protein S (PS), free protein S (fPS), antiplasmin (AP), and plasminogen (PL) were investigated.

For all tests, venous blood samples were collected from both the patient and control groups in the morning after a 12 hour fasting into vacutainer tubes. The coagulation tests were performed on the calibrated daily using kits each of which was compatible with the device.

### 2.3. Statistical Analysis

 Statistical analysis of the data was performed using SPSS for Windows 11.5 software package. The distribution of continuous variables was tested for normality with the Shapiro and Wilk test.

 The significance of the mean difference between the hypothyroid group and the control group was assessed using Student's *t*-test whereas the significance of the median difference between the hypothyroid and control groups was assessed by the Mann-Whitney *U* test. Pearson's chi-square test was used to analyze categorical variables.

 The Dependent *t*-test or the Wilcoxon Signed-Rank test was used to assess the presence or absence of a statistically significant change between baseline and final measurements of the patients in the case group.

 Spearman's correlation test was used to determine the relationship between continuous variables. Multivariate Linear Regression analysis was used to identify the clinical variables most associated with the changes of thyroid hormones. Variables with *P* < 0.25 in the univariate analyses were entered in the multivariate regression model and were considered candidate variables. Multivariate linear regression analysis was performed to determine whether the most significant clinical variables identified by the gradual regression analysis maintained their significant effects on the changes of thyroid hormones after adjustment for diagnosis, age, sex, and body mass index SDS values. The regression coefficient and 95% confidence interval were calculated for each variable. As thyroid hormones did not show normal distribution logarithmically converted data were used in the regression analyses. A *P* value of <0.05 was considered statistically significant.

## 3. Results

### 3.1. Demographic Characteristics

 The study was conducted on 54 children with hypothyroidism and 55 healthy children. There was no difference between the patient and control groups regarding demographic characteristics such as age, sex, height, weight, body mass index (BMI), height standard deviation score (SDS), weight SDS, and BMI SDS (Table 1).

### 3.2. Thyroid Function Tests

 The comparison of basal thyroid function tests between the patient and control groups showed significantly higher TSH in children with hypothyroidism; however, TT4 and fT4 were found to be low (Table 1).

 In the evaluation of posttreatment changes of thyroid hormones in hypothyroid patients, there was a statistically significant decrease in TSH levels (*P* < 0.001), whereas TT4 and fT4 levels were found to be significantly increased (*P* < 0.001 and <0.001, resp.) (Table 2).

### 3.3. Coagulation Tests, Procoagulant and Anticoagulant Proteins

 At the diagnosis, unlike healthy control group, significant decreased results were found in children with hypothyroidism in terms of fibrinogen, TT, and anticoagulant proteins including AT, PC, PS, and fPS (Table 3).

 However, significant improvement was found with respect to APTT, fibrinogen, and TT in children with hypothyroidism (Table 4). Consistent with these findings, significant increase was found with respect to vWF, FVIII, AT, PC, PS, and fPS, during the evaluation of post-treatment changes in children with hypothyroidism (Table 4).

 Spearman's correlation test was used to determine the relationship between thyroid function tests and the study variables. A positive correlation was found between fT4 and vWF, FVIII, PC, and PS (*P* = 0.026 and *r* = 0.21; *P* = 0.01 and *r* = 0.25; *P* < 0.001 and *r* = 0.43; *P* = 0.012, and *r* = 0.24). Multivariate linear analyses showed a negative correlation between TSH and AT (*P* < 0.001 and a confidence interval between −0.044 and −0.016), whereas there was a positive correlation between fT4 and AT (*P* = 0.03 and a confidence interval between 0.0005 and 0.01).

## 4. Discussion

 Thyroid hormones affect all systems and metabolic events in the body in different ways. The effects of hypothyroidism on the coagulation system are still controversial. Although various studies have reported a hypercoagulable state, some other studies have found a hypocoagulable state in hypothyroid patients [3]. Previous studies have demonstrated that adult hypothyroid cases show a prolonged aPTT and a normal or slightly shortened PT in baseline coagulation tests [2, 4, 5].

 Most of the data concerning the effects of hypothyroidism on the coagulation system are based on previous studies, particularly on adult patients with severe hypothyroidism [2, 6–8]. In these studies significantly lower values have been found in vWF, FVIII, FIX, FX, and FXI activities in patients with hypothyroidism. A negative correlation has been also found between TSH and FX activity. These studies also suggested that the decrease in coagulation factors in hypothyroid patients had associated with a general decrease of the plasma protein synthesis. Homoncik et al. [9] reported that vWF levels and FVIII activity were lower in patients with severe hypothyroidism compared to the control group. In the same study, the authors reported that vWF levels and FVIII activity were increased after hypothyroidism treatment. Contrary to these findings, there are studies in the literature reporting that FVIII activity and vWF levels were normal [10] and even increased [11] in patients with hypothyroidism.

 There is a limited number of studies in the literature that examine the coagulation system in children with hypothyroidism. However, to the best of our knowledge, there is only one study evaluating coagulation tests in children [12]. In this study, 22 children with congenital hypothyroidism had been evaluated and a slightly prolonged aPTT had been found only in one child who had been diagnosed with vWD.

 Thus, our study is important for examining in detail the coagulation system in these patients. Significant decreased results were found in children with hypothyroidism in terms of fibrinogen, TT, FVIII, and vWF. In the evaluation of post-treatment changes in a statistically significant increase was found in fibrinogen, TT, vWF, and FVIII. Close relationship between thyroid function tests and the study variables were found. A positive correlation was found between fT4 and vWF, FVIII.

 It has been known that decreases in the activity of anticoagulants AT, PC, PS, and fPS may lead to thromboembolic events. These proteins have also been evaluated within the scope of the relationship between hypothyroidism and hemostasis in previous studies. In a study of 20 hypothyroid patients, Erem et al. [2] found that PC and PS activities were similar between the hypothyroid and control groups, whereas AT activity was higher in hypothyroid patients. In our study, AT, PC, PS, and fPS activities were lower than the healthy controls. This finding is consistent with that of Rennie et al. indicating a lower AT activity [4]. In another study of patients with subclinical hypothyroidism, Müller et al. [3] found that AT, PC, PS and fPS activities were similar between the patient and control groups. However, in our study, AT, PC, PS, and fPS activity had been found to be lower in children with hypothyroidism compared to the controls. These findings suggested that anticoagulant proteins are more affected by thyroid hormones. These findings indicate that there is a general decrease in anticoagulant proteins in hypothyroid children and thrombosis risk may be associated with multiple factors. Significant changes in anticoagulant proteins after treatment suggested that thyroid hormone replacement.

 In conclusion, we would like to emphasize that the coagulation system especially vWF and FVIII, and particularly the anticoagulant system, should be monitored closely in patients followed up for hypothyroidism. Thyroid hormones should be examined and, if necessary, hormone replacement therapy should be administered in patients followed up for a predisposition to coagulation. Additionally, further studies with larger series are needed to investigate the effects of hypothyroidism on the coagulation system.

## Figures and Tables

**Table 1:** Characteristics of the patients and controls.

Characteristics	Patients (*n* = 54)	Controls (*n* = 55)	*P*
Age (month)	78.5 (0.4–186.5)	80.5 (0.4–182.0)	0.709^†^
Sex			0.776^‡^
Female (*n*, %)	27 (50.0)	26 (47.3)	
Male (*n*, %)	27 (50.0)	29 (52.7)	
Height (cm)	107.6 ± 42.8	111.1 ± 42.8	0.667^∗^
Height SDS	−0.34 ± 1.32	−0.20 ± 1.17	0.575^∗^
Weight (kg)	25.8 ± 20.5	25.5 ± 18.6	0.944^∗^
Weight SDS	−0.02 ± 1.40	−0.22 ± 1.13	0.426^∗^
BMI	17.6 ± 3.9	17.1 ± 3.0	0.507^∗^
BMI SDS	0.04 ± 1.41	−0.14 ± 1.34	0.493^∗^
TSH (mIU/L)	8.6 (1.81)	2.4 (1.89)	<0.001
TT4 (*μ*g/dL)	6.1 (3.04)	8.8 (2.80)	<0.001
fT4 (ng/dL)	0.6 (0.20)	1.1 (0.23)	<0.001

BMI: body mass index; fT4: free thyroxine; SDS: standard deviation score; TSH: thyroid-stimulating hormone; TT4: total thyroxinee. ^∗^Student's *t*-test, ^†^Mann Whitney *U*, ^‡^Pearson's chi-square test.

**Table 2:** Thyroid function tests of the patients before and after treatment.

Variables	Before	After	*P*
TSH (mIU/L)	8.6 (1.81)	1.9 (2.13)	<0.001^∗^
TT4 (*μ*g/dL)	6.1 ± 3.04	9.0 ± 1.95	<0.001^†^
fT4 (ng/dL)	0.6 (0.20)	1.13 (0.31)	<0.001^∗^

fT4: free thyroxinee; TSH: thyroid-stimulating hormone; TT4: total thyroxine. ^∗^Wilcoxon testi, ^†^ *t*-Test.

**Table 3:** Data on baseline coagulation tests, pro-coagulant and anti-coagulant proteins in the patients and controls.

Variables	Patients (*n* = 54)	Controls (*n* = 55)	*P*
PT (sc)	11.1 (1.22)	11.1 (1.00)	0.643^∗^
aPTT (sc)	34.9 ± 4.98	35.9 ± 5.12	0.321^†^
INR (ratio)	1.0 (0.11)	1.0 (0.09)	0.662^∗^
Fibrinogen (mg/dL)	244.1 ± 81.35	292.5 ± 81.02	0.039^†^
TT (sc)	19.4 (3.20)	14.1 (2.60)	0.029^∗^
FII (%)	102.7 (30.25)	114.5 (23.60)	0.123^∗^
FV (%)	114.6 ± 31.60	123.8 ± 22.07	0.080^†^
FVII (%)	84.0 ± 20.83	84.1 ± 17.99	0.983^†^
FVIII (%)	82.2 (43.50)	91.5 (38.00)	0.028^∗^
FIX (%)	95.6 ± 29.28	94.5 ± 31.86	0.860^†^
FX (%)	100.7 ± 25.87	109.9 ± 25.04	0.063^†^
FXII (%)	121.2 ± 30.13	122.2 ± 34.84	0.875^†^
FXII (%)	131.5 ± 27.70	120.3 ± 35.87	0.072^†^
AP (%)	119.6 ± 18.42	124.1 ± 14.02	0.155^†^
vWF (%)	105.1 (66.35)	93.4 (61.80)	0.025^∗^
AT (%)	98.5 ± 28.22	115.3 ± 26.38	0.002^†^
PC (%)	81.5 (52.75)	92.0 (58.00)	0.022^∗^
PS (%)	73.4 ± 24.63	83.2 ± 20.10	0.026^†^
fPS (%)	84.1 ± 18.10	100.9 ± 20.31	<0.001^†^
PL (%)	91.0 (44.00)	99.0 (37.00)	0.184^∗^
APCR (R)	3.0 (0.40)	2.9 (0.40)	0.127^∗^
LA (R)	1.0 (0.18)	1.1 (0.17)	0.189^∗^

AP: antiplasmin; AT: antithrombin; F: factor; fPS: free protein S; PC: protein C; PL: plasminogen; PS: protein S; PT: prothrombin time; aPTT: activated partial thromboplastin time; TT: thrombin time; vWF: von Willebrand's factor. ^∗^Mann Whitney *U*, ^†^Student's *t*-test.

**Table 4:** Coagulation test results of the patients before and after treatment.

Variables	Before	After	*P*
PT (sc)	11.7 (1.60)	11.6 (1.20)	0.264^∗^
aPTT (sc)	35.1 ± 5.48	31.6 ± 2.68	0.006^†^
INR (ratio)	1.1 (0.15)	1.1 (0.11)	0.264^∗^
Fibrinogen (mg/dL)	274.9 ± 68.36	303.6 ± 50.50	0.03^†^
TT (sc)	19.5 (3.25)	15.6 (1.40)	0.007^∗^
FII (%)	102.7 (25.15)	111.4 (19.60)	0.245^∗^
FV (%)	105.6 (31.90)	118.2 (24.30)	0.054^∗^
FVII (%)	82.2 ± 20.63	80.3 ± 18.16	0.630^†^
FVIII (%)	87.0 (43.70)	106.5 (32.50)	0.031^∗^
FIX (%)	104.4 (27.95)	109.6 (34.20)	0.068^∗^
FX (%)	99.5 ± 24.89	107.7 ± 20.43	0.110^†^
FXII (%)	128.3 ± 29.20	126.4 ± 23.22	0.750^†^
FXIII (%)	132.8 ± 25.40	140.2 ± 38.58	0.331^†^
AP (%)	121.5 (28.00)	112.0 (10.50)	0.026^∗^
vWF (%)	92.4 (63.60)	106.4 (41.10)	0.025^∗^
AT (%)	92.0 (30.00)	107.0 (18.50)	0.028^∗^
PC (%)	83.0 (40.00)	92.0 (24.00)	0.036^∗^
PS (%)	71.5 ± 21.33	87.7 ± 15.78	<0.001^†^
fPS (%)	80.4 ± 17.94	92.7 ± 19.58	0.003^†^
PL (%)	93.0 (31.50)	90.0 (16.50)	0.439^∗^
APCR (ratio)	3.0 (0.35)	2.6 (0.55)	0.421^‡^
LA (ratio)	1.1 (1.14)	1.2 (1.13)	0.41^‡^

AP: antiplasmin; AT: antithrombin; F: factor; fPS: free protein S; PC: protein C; PL: plasminogen; PS: protein S; PT: prothrombin time; PTT: activated partial thromboplastin time; TT: thrombin time; vWF: vonWillebrand's factor. ^∗^Mann Whitney *U* test, ^†^Student's *t*-test, ^‡^Wilcoxon signed rank test.

## Abbreviations

AP: Antiplasmin
aPTT: Activated partial thromboplastin time
AT: Antithrombin
BMI: Body mass index
F: Factor
fPS: Free protein S
fT4: Free thyroxine
PC: Protein C
PL: Plasminogen
PS: Protein S
PT: Prothrombin time
SDS: Standard deviation score
TSH: Thyroid-stimulating hormone
TT: Thrombin time
TT4: Total thyroxine
vWF: vonWillebrand's factor.
